# Emotional Intelligence and Mental Health in the Family: The Influence of Emotional Intelligence Perceived by Parents and Children

**DOI:** 10.3390/ijerph17176255

**Published:** 2020-08-27

**Authors:** María Trinidad Sánchez-Núñez, Noelia García-Rubio, Pablo Fernández-Berrocal, José Miguel Latorre

**Affiliations:** 1 Department of Psychology, Faculty of Education, University of Castilla La Mancha, 02071 Albacete, Spain; 2 Department of Economics and Statistics (DHEP), Faculty of Economics and Business, University of Castilla La Mancha, 02071 Albacete, Spain; noelia.garcia@uclm.es; 3 Department of Basic Psychology, Faculty of Psychology, University of Málaga, 29071, Málaga, Spain; berrocal@uma.es; 4 Department of Psychology, Faculty of Medicine, University of Castilla La Mancha, 02006 Albacete, Spain; jose.latorre@uclm.es

**Keywords:** self-reported emotional intelligence, perceived emotional intelligence, mental health, family, parents, mothers, children, adolescents

## Abstract

Introduction: The relevant scientific literature has confirmed the relationship between emotional intelligence (EI) and mental health. In addition, previous studies have underlined the importance of perceived EI between family members in the construction of one’s own EI. Adolescence is considered to be a crucial stage in identity construction and a time when mental health is vulnerable. Objectives: To analyze the mediating role of self-reported EI on mental health of adolescents and young adults still living in the family home, we considered the relationship between perceived EI in parents and children. Method: The sample was comprised of 170 children and their respective fathers and mothers living in the same family home. Self-reported EI was evaluated using the Trait Meta-Mood Scale (TMMS-24), whereas perceived EI was evaluated via the Perceived Emotional Intelligence Scale-24 (PTMM-24) and mental health using the MH-5. Results: Parents’ perceived EI of their children also children’s perceived EI of their parents has a direct effect on children’s mental health and an indirect effect through the EI self-reported by children. We discuss the differences in the role of mothers and fathers in emotional education and its influence on the results. Conclusions: We highlight the importance of perceived EI among family members, over and above the self-reported EI of each member, for its predictive power on the mental health of children.

## 1. Introduction

The concept of emotional intelligence (EI) has been defined as “the ability to perceive accurately, appraise, and express emotion; the ability to access and/or generate feelings when they facilitate thought; the ability to understand emotion and emotional knowledge; and the ability to regulate emotions to promote emotional and intellectual growth” [1] (p. 10). The field of EI assessment has proposed different instruments, depending on whether to evaluate these emotional abilities using self-assessment scales, such as the TMMS-24 [2], or ability measures, such as the Mayer–Salovey–Caruso Emotional Intelligence Test (MSCEIT 2.0) [3].

Self-reports are a subjective measure to evaluate EI since they assess the perception of our EI [1,4]. Self-assessment measures have been shown to have a significant relationship with key criteria variables of mental health, psychological adjustment, and well-being [2,5,6,7,8,9].

In the Spanish-speaking population, one of the most widely used instruments to evaluate self-reported EI is the TMMS-24 [10]. This scale evaluates the perception that each person has of their level of attention, clarity, and repair on emotions, revealing the existence of a differential profile in its components [6]. People with high EI are characterized by moderate to low scores on emotional attention and high scores on clarity and repair [11]. Higher levels of clarity and repair are associated with better psychological adjustment and mental health [12,13,14]. On the other hand, people who attend to their emotions excessively, without adequate clarity and repair, can develop an emotional spiral that may generate a ruminant process. This does not allow for the causes, motives, and consequences of these emotions to be understood, which, in turn, would maintain, rather than remedy, a negative emotional state [13,15]. Deficits in emotional clarity have been linked to symptoms of anxiety and stress, and when accompanied by deficits in repair, have been linked to symptoms of depression [13,14,16]. Of the three factors of EI assessed through TMMS-24, repair is the most appropriate component for predicting psychological adjustment indicators [6].

In short, the relationship between EI and mental health has been demonstrated through several meta-analyses [8,17,18] and extensive reviews [5,19]. The research in this field concludes that people with mental health problems have a significantly lower level of EI than the general population. In the opposite sense, there is some preliminary evidence to suggest that some forms of EI may protect people from stress and lead to better adaptation [20,21].

Mental health in adolescence has come to be considered a public health problem and studies in this area are aimed at preventing and promoting mental health in this life stage [22,23]. Some studies exploring the relationship of emotional intelligence with mental health among early adolescents have found a significant relationship between emotional intelligence and scores on mental health scales and sub-scales [24]. Shabani and Damavandi [25] found that students’ mental health could be predicted by EI, with the EI predictor variable explaining about 35.7% of the variance in mental health. Other studies have revealed a significant relationship between EI and emotion management and mental health problems in adolescents [26]. Adolescence is a critical period and requires development of emotional regulatory skills.

### Emotional Intelligence in the Family and Mental Health

It is important to identify the variables associated with mental health indicators, such as emotional intelligence, once adolescence has been reached. However, we also consider it essential to explore the origin and possible mediators of the association between EI and mental health during this life stage. With regard to the socialization of EI, the model proposed by Zeidner et al. (2003) underlines the importance of genes, the family environment, culture, and other socialization agents during EI development [27]. Studies in the field of genetics, although scant, provide further empirical support for the conceptualization of EI as a personality trait [28]. Nonetheless, it is important to explore other factors that shape EI traits and which might be managed or modified when improving and developing IE, and thus, the well-being of future generations.

The ways in which culture is infused in every aspect of life mediates the roles the different family members are expected to adopt [29]. The effects of this on developing infants’ emotional experience [30] and on adolescents’ EI [31] have also been evidenced. In addition, focusing now on the family setting, parents’ EI has an impact on the emotional learning of their children [32,33]. The family system is where children learn when and how to express feelings, manage conflict, and negotiate differences, where healthy interactions between youths and family help develop EI traits [34]. There is a relationship between the self-reported EI of parents and children [35,36], and parenting and children’s EI traits [37]. There are two socialization routes through which parents may teach their children emotional intelligence competencies [27,38]:

(a) The direct effect route, through which parents explicitly transmit emotional abilities to their children. For example, through explicit or informal lessons, conversations on emotional regulation, direct training, coaching, guidance, or reinforcement of expressive behavior.

According to Salovey et al. [39], parents who are emotionally sensitive to their children’s emotional needs usually have emotionally intelligent children. Research confirms the association between parents’ level of emotional control or regulation and that of their children [40]. Parents who are able to explain and reflect on their own and their children’s emotions help them to develop strong ‘mentalizing’ capacities in which emotions are understood and skillfully regulated [41]. Other studies have shown that parents’ positive expressiveness (mainly in mothers) mediates the relationship between parental warmth and empathy in children, and that, in turn, children’s empathy mediates the relationship between parental positive expressiveness and children’s social functioning [42]. Thus, a bidirectional model of effects between the emotional conduct of parents and children is evidenced [43,44].

(b) The indirect effect route, through which parents transmit emotional abilities to their children implicitly or unconsciously. For example, through the observation and modeling of the competencies and emotional responses of others.

Parental attributions, beliefs, and expectations may have considerable consequences for the way in which parents socialize their children [45]. It is worth highlighting the importance of this indirect effect route in the instruction and development of emotional abilities as it acts at an implicit, unconscious, or hidden level. There are few studies on how parents perceive their children’s EI and the influence they have on their own EI. In a study conducted by Sánchez-Núñez et al. [35] in a Spanish sample, self-reported EI by teenage children was largely predicted by the parents’ perception of children in EI and vice versa, rather than by the EI self-reported by parents and children. Further, parents and children perceived each other’s EI in a statistically significant similar way in the three factors affecting EI. Parents perceived their children’s EI similarly to how they perceived themselves, whereas children perceived their parents correlatively to how they perceived themselves and how their parents perceived them. There is a similar phenomenon in the field of general intelligence. In a study conducted by Furnham et al. using intelligence quotients (IQ), parents’ estimate of their own intelligence correlated with, and even had predictive power for, their estimate of their children’s intelligence [45,46]. In the same line, a two-tailed bivariate correlation analysis with parental estimates of children’s overall IQ (g) and children’s standardized mean cognitive ability score as the variables revealed a statistically significant positive relationship [47].

These results are useful in the field of family intervention, since, as previously demonstrated in the scientific literature, unstable parents tend to project their problems onto their children and create inadequate expectations [48]. These expectations could generate maladaptive perceptions of their emotional abilities and promote mental health problems. In fact, depressive symptoms are associated with both more accurate and more biased interpersonal perceptions [49]. If we wish to project an emotionally stable image on children and help develop their emotional balance, parents should attempt to be emotionally balanced. The judgments children hear about themselves determine how they perceive themselves and this typically begins to occur around the age of two [50], increasing in adolescence, when the construction of identity through the perception of their parents, among other agents of socialization, is key. However, some studies have demonstrated the influence that children’s perception of parenting styles in their family could have on their emotional development in relation to self-esteem, depression, or anxiety [51,52], as well as the influence of specific cognitive appraisals and discrete emotional states [53]. Adolescents with more serious mental health problems perceive their families as less emotionally involved and more critical, and as having a lower level of emotional intelligence [26]. In the same way, perceived family acceptance is seen as a marker of vulnerability, shown by the detrimental consequences of substance use and the risk of substance use disorders, as well as adolescent maladjustment [54].

Regarding parents’ gender, the different roles our society attributes to mothers and fathers involves mothers having greater responsibility than fathers for the care and emotional education of their children [29,30]. In this respect, women may be more closely and deeply connected to others throughout their life courses [55,56] and specifically to their children, in terms of the parent-child relationship, and have stronger, more frequent, and more reciprocal ties to children than men [57,58]. The close relationship between emotional competencies and women from an early age has been attributed to socialization that involves more contact with feelings and their nuances. They are viewed as typically more emotionally expressive than men, with greater emotional understanding and recognition of emotions in others, being more perceptive and empathic [59,60]. In the family environment, these assertions were corroborated in a study carried out with a Spanish sample where mothers showed a greater perceptual adjustment of their children’s EI than fathers [61].

No studies have demonstrated the mediating role of children’s EI in the relationship between the perceived EI between family members and the mental health of adolescent children. In pursuit of better ways to improve emotional intelligence in adolescents, we delved deeper into the routes of family socialization. Specifically, we aimed to discover the implicit routes that influence and interact with adolescents’ mental health including their perceptions of family emotional intelligence. It is important to identify factors that may mediate this relationship in order to implement appropriate interventions in this area. Not only do we consider self-reported emotional intelligence (EI) in children but also their perceived emotional intelligence (PEI) of their parents and vice versa. This type of family analysis will provide unique information that is lacking in the body of documented data on mental health and minimizes the social desirability effect inherent in self-report measures. The main aim of this study was to determine to what extent the mental health of adolescents and young adults who still live in the same family home is determined by their EI, the perception of the EI of their parents, and the parents’ beliefs about their own emotional abilities. Thus, we highlighted the complexity of such an analysis, considering the multiple factors involved and the many possible relationships that may be found between them; a field as yet unexplored in the literature. Our intention was to explore whether the perceived EI among the members of a family might impact on the mental health of adolescent sons and daughters, and the meditating role of the children’s self-reported EI in this relationship. We thus opened up a new perspective in the study of EI, underlining the role that routes of indirect socialization may have on the development of adolescents’ EI and their mental health. To this end, and based on the literature reviewed, the following hypotheses (Note that the direction of the possible relationships has not been hypothesized given the complexity of the problem resulting from not being able to consider a single measure of EI, but rather the three factors or subscales) were raised:

**Hypothesis 1** **(H1).** *Self-reported EI in children plays a mediating role in the relationship between the EI perceived by parents in their children and the children’s mental health.*

**Hypothesis 2** **(H2).** *Self-reported EI in children plays a mediating role in the relationship between the EI perceived by children in their parents and the children’s mental health.*

**Hypothesis 3** **(H3).** *Mothers exert a greater influence, attributed to a socialization that involves more contact with feelings, than fathers on their children’s mental health through mutually perceived EI.*

**Figure 1:**
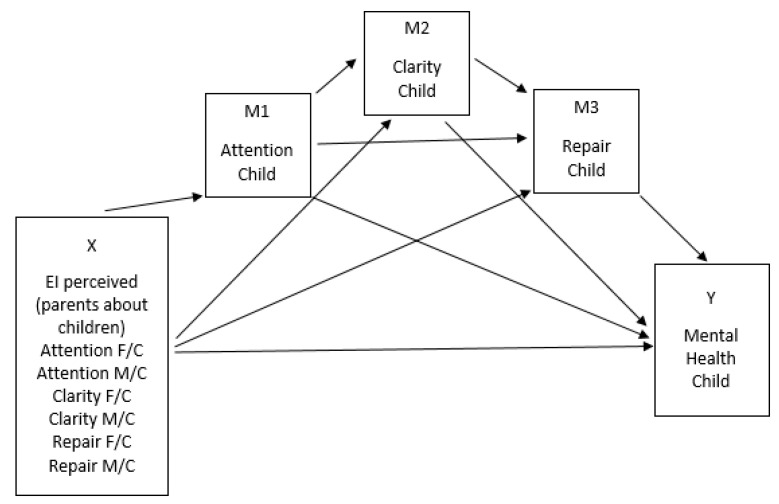
Hypothesized model (H1), (F/C = Fathers’ perceived emotional intelligence (EI) of their children; M/C = Mothers’ perceived of their children).

**Figure 2:**
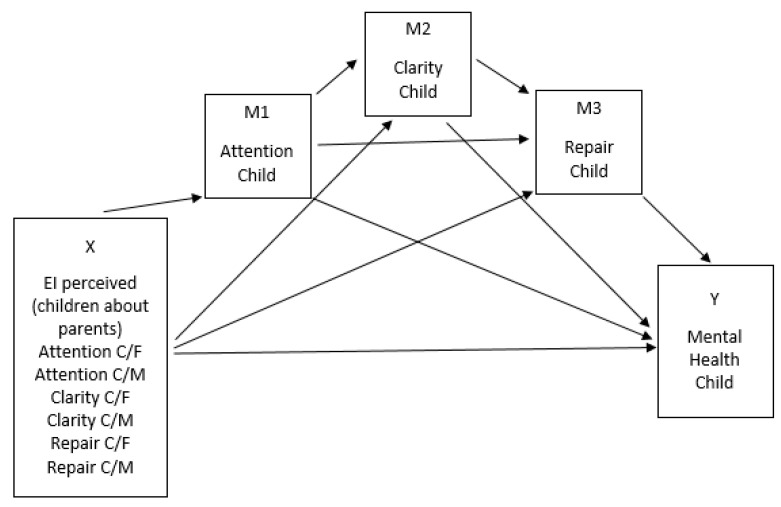
Hypothesized model (H2), (C/F = Children’s perceived EI of their fathers; C/M = Children’s perceived EI of their mothers).

Figure 1 shows the model as hypothesized by H1, i.e., the mental health of children (Y) would be explained by the attention, clarity, and repair reported by the father and mother as their perceptions about their children (X’s), not only directly but also through the children’s self-reported EI (serial mediators, M’s). Figure 2, in turn, shows the model as hypothesized by H2, i.e., the mental health of children (Y) would be explained by the attention, clarity, and repair reported by the children as their perceptions about their father and mother (X’s), not only directly but also through the children’s self-reported EI (serial mediators, M’s).

## 2. Materials and Methods

### 2.1. Participants

The sample was comprised of 170 adolescents and young adults (85 male and 85 female) and their respective parents. Participants were recruited among students of the University of Castilla-La Mancha (Spain). The sample was expanded with students’ siblings, over the age of 14, and who lived in the same family unit. The age range of the participants was between 14 and 34 years (M = 21.50; SD = 3.73). Participation was voluntary. The questionnaires were completed privately and anonymously at the family home.

### 2.2. Instruments

The evaluation instruments used in this study were the following:

The Trait Meta-Mood Scale-24 (TMMS-24) [10] is a scale comprised of 24 items and is intended to measure the participant’s perceived EI. The respondents are required to indicate their level of agreement with each item on a five-point Likert type scale from strongly disagree (1) to strongly agree (5). The scale is made up of three factors or sub-scales: attention to feelings, emotional clarity, and mood repair. Attention to emotions, measured through 8 items, is how much attention individuals believe they pay to their inner feelings (“I think about my mood constantly”). Clarity, measured through the next 8 items on the scale, is how individuals believe they perceive their emotions (“I am often confused about how I feel”). Finally, repair, measured by the last 8 items, is the individual’s belief of their ability to terminate negative emotions and prolong positive ones (“Although I am sometimes sad, I have a mostly optimistic outlook”).

The relationship pattern of EI factors shows a positive relationship between attention and clarity, and clarity and repair, but a negative relationship between attention and repair [6]. Persons with a high EI in the Spanish-speaking population have a model characterized by moderate to low scores in attention and high scores in clarity and repair [6,13,14].

The authors of this scale found an internal consistency of 0.90 for attention 0.90 for clarity and 0.86 for repair and that it improved the psychometric properties of the longer 48-item version (0.86 for attention, 0.87 for clarity, and 0.82 for repair) [2]. The indirect reliability indicator for this scale, measured by Cronbach’s Alpha, was satisfactory, with internal consistencies for attention 0.88, clarity 0.89, and repair 0.86.

The Perceived Emotional Intelligence Scale-24 (PTMMS-24) [35] is a version of the TMMS-24 developed by Fernández-Berrocal et al. [10], specifically adapted for this study. The PTMMS-24 evaluates parents’ PEI of their children and children’s PEI of their parents in the three factors or sub-scales: attention to your own feelings, emotional clarity, and repair of emotions. For this reason, the 24 items of the scale are written in third person. The parents are asked to think about their son/daughter when scoring the items and the sons/daughters are asked to think about their father/mother. Attention to emotions, measured through 8 items, is how much attention parents believe their children pay to their inner feelings and vice versa (“He/She thinks about his/her mood constantly”). Clarity, measured through the next 8 items on the scale, is how parents believe their children perceive their emotions and vice versa (“He/She is often confused about how he/she feels”). In addition, repair, measured by the last 8 items, is the parent’s belief of the ability of their children to terminate negative emotions and prolong positive ones and the same for the children and their parents (“Although he/she is sometimes sad he/she has a mostly optimistic outlook”).

This allows the self-reported measures to be compared with those reported by the rest of the family members on the subject in question. We believe this will provide interesting information on the intrapersonal and interpersonal perception of emotional abilities among family members. The indirect reliability indicator for this scale, measured by Cronbach’s Alpha, was satisfactory, with internal consistencies of above 0.80 for each self-reported EI sub-scale (Attention 0.86; Clarity 0.87 and Repair 0.88).

Mental Health-5 (MH-5) [62], adapted to Spanish by Alonso et al. [63], is composed of 5 items in the area of emotional well-being and assesses subjects’ mental health, which is measured as the degree of depressive and anxious symptomatology that the subject has presented in the past month. A high score on this scale is associated with better mental health. The brevity of the scale makes it a useful instrument for research. The answers are coded using a Likert scale ranging from 1 = always to 6 = never (always, almost always, often, sometimes, rarely, never). Participants are asked questions such as: “During the past four weeks, how often were you feeling so down that no one could cheer you up?” Its psychometric properties have shown adequate reliability and validity over more than a decade of ongoing research. Studies that have used this scale show adequate scale reliability, with Cronbach alpha ranging between 0.77 and 0.85 [64]. In our study, the indirect reliability indicator for this scale, measured by Cronbach’s Alpha, was satisfactory, with internal consistencies for children’s MH-5 of 0.85.

### 2.3. Procedure

The eligible population was university students enrolled in the first year of study and their brothers and sisters over the age of 14 sharing the family unit. Participants who had already left the family home were excluded. The participation of the subjects was voluntary and the tests were completed privately and anonymously in the family home. A letter of consent and information about the study and its procedure was distributed to all family members through the students (children). The assessment instruments were given to students with an identification code and an individual envelope for each family member, which they had to deliver closed and sealed to protect the privacy and confidentiality of their relatives’ responses. The questionnaires did not collect information that could damage the mental or social integrity of the participants.

### 2.4. Statistical Analysis

The statistical analysis was conducted using IBM SPSS Statistics 24. First, the variables included in the study were analyzed univariately by means of their main descriptive statistics and, bivariately, through Pearson’s correlation matrix.

The main analysis consisted of testing two mediation models. A simple mediation model is intended to demonstrate how a variable’s effect (X) on an outcome (Y) can be partitioned into direct and indirect effects that can be quantified using OLS regression. The presence of such indirect effects assumes there is an intervening variable or mediator (M) that is, at the same time, a consequent variable for X and an antecedent variable for Y [65].

In order to analyze the intergenerational pathways through which bidirectional perceptions influence adolescents’ self-reported EI and how this influences their mental health, the mediation model is an appropriate statistical instrument. Therefore, the mediators here are represented by the children’s self-reported EI variables. Moreover, considering that the three sub-constructs of EI are considered to be sequentially related, i.e., attention can influence clarity and clarity can influence repair, the serial mediation model is preferred to the parallel one, since it is able to capture this chain of relationships. This model allows us to study both the direct effect, represented by a direct path from the input to the output variables, and the indirect effects (paths through a single or chained mediators)

These effects could be summarized as follows:

Direct effect: X’s→Y

Indirect effects: X’s→Attention→Y; X’s→Clarity→Y; X’s→Repair→Y;

X’s→Attention→Clarity→Y; X’s→Attention→Clarity→Repair→Y; X’s→ Clarity→Repair→Y,

being X’s the EI perceived by father/mother about their children (model under H1) and EI perceived by children about their father/mother (model under H2), and Y being the children’s mental health.

To test the mediation models, the available macro for SPSS, known as PROCESS v 3.4, was used.

## 3. Results

Table 1 shows the main descriptive statistics (mean and standard deviation) and the Pearson correlation coefficients for the variables involved in the statistical analysis.

As can be seen in Table 1, children’s mental health correlates inversely with their self-reported attention and directly with repair. With relation to the EI perceived by the parents about their children, only one significant correlation can be found, i.e., children’ mental health correlates inversely with the children’s attention perceived by their mothers. However, if we consider the EI informed by the children about their parents, children’s mental health correlates positively with clarity and repair, but only for fathers.

### Multivariate Analysis

The first serial multiple mediator model tested included six explanatory variables (X’s), collecting the EI perceived by fathers and mothers about their children (attention F/C and M/C, clarity F/C and M/C, and repair F/C and M/C). The outcome variable (Y) was children’ MH-5 and three serial mediators (M’s) were considered to link X and Y. These mediators represented the self-reported EI (attention C, clarity C, and repair C). The order in which serial mediators are included is significant because it establishes the causal relationships between them. In this case, attention could influence clarity and repair could be influenced by attention, clarity, and the attention→clarity path. Therefore, three serial mediators result in a total number of 7 possible mediation effects (X’s→M1→Y; X’s→M2→Y; X’s→M3→Y; X’s→M1→M2→Y; X’s→M1→M3→Y; X’s→M2→M3→Y; X’s→M1→ M2→M3→Y). Table 2 shows the coefficients of all sub-models presented. In these sub-models, the outcome variable changes. The first sub-model explains attention C through the perceived EI variables; in the second model, clarity C is explained by means of all the EI perceived variables and attention C (the former mediator) and so on. Table 3 shows the indirect and direct effects, showing the point estimation and the confidence interval based on 10,000 bootstrap samples. The indirect effects can be interpreted as significantly positive (negative) if the bootstrap confidence interval is entirely above (below) zero.

Starting with the mediating role of self-reported attention (attention C), it can be seen that the way fathers perceive attention in their children positively influences their self-reported attention (coeff. = 0.2936; *p* = 0.0007). In turn, the self-reported attention has a negative effect on their mental health (coeff. = −0.2851; *p* = 0.0012). Thus, the indirect effect of how fathers see their children in attention on children’s mental health is significant and negative since the bootstrap confidence interval is entirely below zero (c.i. = (−0.1726; −0.0199)). In this case, the direct effect of attention F/C on mental health is not statistically significant (coeff. = 0.0271; *p* = 0.7808). The mothers’ perception of their children in repair has a significant indirect effect on mental health through self-reported repair. In this case, the effect is positive (c.i. = (0.0007; 0.1508)). As significant direct effects on mental health, only repair perceived by fathers in their children can be highlighted, with the sign being positive (coeff. = 0.2976; *p* = 0.0037).

Table 4 and Table 5 show the results for the model used to study the mediating role of the self-reported EI in the relationship between the way children perceive the EI of their parents and their own mental health. In this case, through the children’s attention as mediator, significant indirect effects can be found for the attention the children perceive in both their mothers (c.i. = (−0.2228; −0.0394)) and fathers (c.i. = (−0.1608; −0.0242)). As stated for the previous model, the sign is negative (the higher the perceived attention, the higher the self-reported attention and the lower the mental health).

Considering repair as mediator, we found the perceived clarity of children on their mothers had a negative indirect effect (c.i. = (−0.1642; −0.0047)). The perception of children about clarity in their mothers had a negative effect on their own repair (coeff. = −0.2533; *p* = 0.0133) and this had a positive effect on mental health (coeff. = 0.2678; *p* = 0.0037). The children’s perception of their mothers in repair showed a significant indirect effect through this mediator, with a positive sign in this case (c.i. = (0.0176; 0.1738)).

Significant effects do arise here when considering serial mediators. Children’s clarity mediates through children’s repair (Mediator 6). The indirect effect of the children’s perception in attention in their fathers is significant and, in this case, is positive (c.i. = (0.0000; 0.0385)). The same result was found for clarity perceived by children on their mothers, also with a positive sign (c.i. = (0.0000; 0.0585)).

As a direct effect on mental health, only the repair perceived by children on their fathers was found to be significant and positive (coeff. = 0.2634; *p* = 0.0029).

## 4. Discussion

This study examined the serial multiple mediation roles of three factors of self-reported EI in adolescents and young adults (who live with their parents) on the relationship between perceived EI between parents and children and children’ mental health. The correlation analysis shows significant relationships between variables such as children’s self-reported repair and mental health and inversely with self-reported attention. These results corroborate previous studies on EI and mental health, where the attention and repair factors usually show the most significant relationships with the criteria variables of well-being or emotional adjustment [2,5,6,7,8,21]. With relation to the EI perceived by the parents about their children, only one significant correlation can be found, i.e., the greater the attention perceived by mothers about their children, the lower is the children’s self-reported mental health. However, if we consider the perceived EI by children about their parents, greater clarity and repair perceived in fathers by the children correlates with higher children’s self-reported mental health. However, the mediation analysis of children’s self-reported EI between the variables under study yields more enlightening results.

### 4.1. Hypothesis 1

Regarding Hypothesis 1, where self-reported EI in children was expected to play a mediating role in the relationship between the EI perceived by parents in their children and their own mental health, the results confirm the hypothesis in the case of both father and mother. In the case of the fathers, the emotional attention perceived in their children has a positive and significant effect on the children’s self-reported attention and this, in turn, has an effect on their mental health but with a negative sign. In short, the greater the level of emotional attention perceived by fathers in their children, the greater is their children’s self-reported emotional attention, which has a negative impact on their mental health. The sign of this relationship between attention and mental health is negative, as in previous studies, where the emotional attention factor, in a Spanish-speaking sample, has a negative relationship with mental health or well-being variables [6,14]. This phenomenon would be explained, as suggested in the previous literature, by the negative effect of paying too much attention to emotions without adequate clarity and emotional repair, encouraging a ruminant process [6,15].

In the case of mothers, the effect occurs through repair, where the mothers’ perception of their children in repair has a significant indirect effect on their mental health through the children’s self-reported repair. The sign of the relationship in this case is positive. In the same direction, but adding a direct effect (without mediating through their children’s self-reported EI), is the influence that fathers exert on their children’s mental health through the emotional repair perceived in them, with this relationship being positive. The relationship between attention, repair, and mental health corroborates the prominence of the repair factor to predict mental health and well-being variables [6,8], but in this case manifested through family members. In short, the greater the emotional repair perceived by mothers in their children, the greater is the children’s self-reported emotional repair and the greater their mental health. In the case of fathers, the influence is direct, i.e., the greater the perception of emotional repair in their children, the greater their children’s mental health.

For emotional clarity, we found no significant relationship. It is important to remember that each of the EI factors functions independently despite being interrelated and develop sequentially across the whole lifespan. Individuals cannot regulate their emotions if first they are not attentive to them and clarify them [2,6,9].

We must highlight that, in relation to this hypothesis or explanatory model, there have been no significant serial effects between the mediating variables studied (attention, clarity, and repair) and the rest of the variables under study, although the results show a significant serial effect between the mediating factors of children’s self-reported clarity and repair. The confirmation of this hypothesis supports the effect of indirect or covert routes of socialization on the development of children’s EI in the family [27], which, in this case, is through the image or perception parents have of their children’s emotional skills and its impact on mental health. Our findings coincide with previous studies on the inadequate expectations that parents project on their children and their impact on mental health [48,49].

### 4.2. Hypothesis 2

Regarding Hypothesis 2, which posits that self-reported EI in children plays a mediating role in the relationship between the EI perceived by children in their parents and their own mental health, the results confirm our hypothesis. A variety of relationships and influences are revealed, taking both parents into account. The perception that children have of fathers and mothers in emotional attention has a direct effect on their own self-reported attention, which in turn negatively affects their mental health.

When considering serial mediators, rather than in parallel, an earlier mediator is allowed to influence a later mediator. In this case, we found a positive indirect effect of the attention that children perceived in their fathers on their own mental health. This effect is manifested through the self-reported clarity of the children and their repair. In other words, increased perceived attention of children in their fathers has a positive effect on children’s self-reported clarity, which in turn positively affects their repair, and ultimately their mental health. This result may seem contradictory to the previous finding, but both cases are an example of the functioning of each one of the three factors of EI, when the others remain constant. Thus, greater children’s perceived attention in parents, when clarity and repair remain constant, produces a negative effect on their mental health. However, if the greater perceived attention of children about fathers (taking into account that men generally pay less attention to their emotions compared to women) [60] influences their development of clarity and repair, the final result would be positive about their mental health.

In relation to the children’s perceived clarity in their mothers, this also exerts a direct negative effect through their own emotional repair. This, in turn, has a positive effect on their mental health, although the overall effect on mental health is negative. The relationship between children’s perceived clarity in mothers and children’s self-report repair in this case is negative, being the exception that contrasts with the previous results on the operation of the factors in EI, where clarity and repair usually show a positive relationship [6].

As was the case of children’ perceived attention about parents, the results for the influence of the children’s perceived clarity about mothers adopt different paths, depending on the constancy of each of the factors of self-reported EI. Thus, when the children perceive the mothers as having high clarity, if their own clarity remains constant, this fact has a negative impact on their repair, which finally makes the overall effect show an indirect negative relationship on their mental health. In another sense, when considering serial mediators, the children’ perceived clarity about mothers can positively influence their own self-reported emotional clarity, which in turn improves their repair and mental health. Both cases are examples of the influence pathways of EI in the family when the factors develop or remain constant. A possible explanation for these results could be the paradoxical effect of the variables under study, given the multiple relationships that can be displayed in a context as complex as the family environment. However, a possible explanation, more specifically, could be based on the interaction between the parents’ gender and the developmental moment of the children’s EI. Regarding gender, previous studies confirm women present more clarity about their emotions and perceive emotional signals more easily [59,60]. If, to this fact, we add that the children are in the process of developing their EI, a high degree of emotional clarity perceived in the mothers would require the children clarify and regulate the emotions that are being clarified, exposed, or evidenced. Therefore, if the development of children’s EI factors were in process and the clarity remains constant, these could be perceived as ineffective. In fact, the effective development of the EI factors occurs over time and in series, with the emotions first being attended to, then later clarified and finally potentially regulated [1,6,39].

In relation to the children’s perceived repair about their parents, it is only the children’s perceived repair in mothers, which has an indirect effect on their own self-reported repair and this, in turn, on their mental health. In this case, repair is enhanced in the prediction, while the fathers lose predictive power. The influence of children’s perception of their mothers’ and fathers’ EI and its direct and indirect impact (through their own EI) on their mental health, further supports the role of the routes of indirect socialization in family EI, in this case, through observation [27].

### 4.3. Hypothesis 3

Regarding Hypothesis 3, which states that mothers will exert a greater influence than fathers on their children’s mental health through mutually perceived EI, the results are confirmed or not depending on whether the influence is determined by the parents’ perception of the children or the children of their parents. Thus, in the case of the perception that parents have about their children’s EI, it is the fathers who show greater prominence in the influence that their perceptions have on the children’s mental health indirectly (through children’s self-reported EI) or directly. In this case, the mothers exert their influence only through the repair perceived in their children and indirectly, through their self-reported repair. Since previous studies show the greater perceptual adjustment of mothers on the EI of their children [61], the influence of the perceptions could be lower in the mothers so as to be more adjusted to the reality of how the children perceive themselves. In the case of fathers, the perception of their children in EI exerts an important influence on their self-reported attention and repair, with the latter case having a direct effect on children’s mental health.

In the case of children’s perceived EI about their parents, the opposite occurs; it is the children’s perceived EI in mothers in the three factors that exerts an indirect influence on their mental health through their own three factors of EI. In this line, parents only exert their influence through the degree of emotional attention that children perceive in them. A possible explanation for these results may arise from matriarchy or patriarchy patterns. The role of the father and mother in the development of their children’s emotional abilities may exert a differential influence that causes the children to focus more on certain behaviors in mothers, perhaps because they are more skilled or more involved in emotional education [57,60,61]. On the other hand, the dominant role of the father, as patriarch, can exert a great influence on his children through the image he projects on their own emotional abilities.

Deeper research on the subject is required; studies in this regard are scarce and collecting data within the family framework is typically complicated. On the other hand, including more objective assessment instruments for EI, such as the MSCEIT [3,4], will facilitate the study of such influences through the emotional intelligence measures executed, as previous studies on intellectual quotient have done [45,46]. In this way, we could evaluate the impact that the aforementioned indirect socialization pathways in EI could have on both self-reported and executed EI.

It should be noted this study has some limitations that mean the results should be generalized with caution. Sample size and age range may have conditioned the results. However, the aim of this study was to determine the degree of influence of the indirect socialization pathways of EI when the children share the family home, since in this environment the necessary interactions that we intended to evaluate are still taking place. As sample recruitment is complicated, in future studies, it would be advisable to replicate the findings with a greater age range and a larger sample size.

## 5. Conclusions

The findings of this study lead us to confirm the hypotheses raised. In relation to the first hypothesis, the perception of parents about their children in some factors of EI exerts an influence on the self-reported EI of their children in the same factors, which ultimately determines their mental health. In this case, fathers exert the greatest influence indirectly through the degree of emotional attention they perceive in their children and directly through the emotional repair perceived in them. In the case of mothers, this influence is only exercised indirectly through the repair perceived in their children.

In relation to the second hypothesis, the results confirm the influence of the perception of children about their parents in the different factors of EI on their own self-reported EI and, in turn, on their mental health. In this case, the mother is the reference figure, since it is the children’s perception of the children about the mothers in the three EI factors that shows an indirect and significant effect on children’s mental health, through their own IE. However, from this perspective, the father only shows this type of influence through the degree of emotional attention that the children perceive in their fathers.

In relation to this predictive model, we must highlight the manifestation of different routes of influence, depending on whether the factors of EI self-reported by the children remain constant or developing, being a manifestation of what could occur in the family environment. When parents are perceived with high attention and mothers with high emotional clarity, children would have the option of adjusting or not through the development of their own clarity and repair, influencing their mental health differentially.

Finally, the third hypothesis focused on the differences between fathers and mothers in relation to the influence that mutually perceived EI (parents on children and vice versa) exerts on their children’s mental health. We can conclude that mothers exert a greater influence on their children’s mental health when the perception is the children’s of their parents. However, in the case of the perception that parents have about their children, fathers have a direct and significant influence on children’s mental health. These results lead us to be cautious about the predominant role of gender in emotional education, given the proven mutual influences, where both fathers and mothers have an influence on their children’s mental health. These results would be interesting for future interventions in preventive or clinical practice in the family, as well as to try to balance the weight of emotional education in both parents.

Extremely interesting results to be highlighted are those concerning the effect of the routes of indirect socialization on the development of EI [27]. We provide evidence for the importance of parents’ perceptions of their children and the influence they exert on their emotional development and mental health, as well as the importance of the image that children forge of their own skills by observing their parents exhibiting these skills in practice, which subsequently affect their mental health. It is important to clarify the role of the judgments or perceptions that parents form regarding their children and those of children with respect to their parents, and the effect this has on key variables like mental health. Family factors plays an important role in adolescent well-being [66]. Another of the conclusions to highlight from this study is the relationship of these results with the manifestation of the well-known Pygmalion Effect, a situation in which a person’s expectations of another person’s behavior is what really helps to achieve that behavior [67] but this time could be manifested in the family environment and referred to the skills in EI.

However, it is worth underlining the importance of exploring the weight of culture and social convictions in gender roles and emotional behavior as well as in the expectations placed on each of the different family members [30]. Among the limitations of this study, we could highlight that the findings should be generalized with caution, as we do not know whether the results would be similar in a broader sample or in a different cultural setting. As a recommendation for future research, it would be interesting to elucidate the weight of culture and the generational effect in these types of relationships.

The aim is for the individual to be able to develop their own EI in an autonomous and healthy way, being aware of the influence of these perceptions in order to manage them and not identify with them. Ultimately, these results highlight the indirect emotional socialization pathways (observation and modeling) reviewed in the development models of EI [27,38] for future therapeutic interventions in family and socio-educational fields with the aim of intervening in the improvement of mental health in adolescence and early adulthood.

## Figures and Tables

**Table 1:** Main descriptive statistics and Pearson correlations.

	1	2	3	4	5	6	7	8	9	10	11	12	13	14	15	16
1. MH5 C	---															
2. Attention C	−0.21 **	---														
3. Clarity C	0.14	0.19 *	---													
4. Repair C	0.33 **	0.06	0.24 **	---												
5. Attention F/C	−0.07	0.34 **	0.23 **	−0.06	---											
6. Attention M/C	−0.17 *	0.29 **	0.13	−0.05	0.37 **	---										
7. Clarity F/C	−0.01	0.12	0.16 *	−0.06	0.48 **	0.21 **	---									
8. Clarity M/C	−0.00	0.19 *	0.23 **	0.10	0.27 **	0.51 **	0.38 **	---								
9. Repair F/C	0.13	0.15 *	0.06	−0.06	0.32 **	0.16 *	0.60 **	0.28 **	---							
10. Repair M/C	0.07	0.07	0.07	0.16 *	0.08	0.31 **	0.12	0.49 **	0.28 **	---						
11. Attention C/F	0.08	0.35 **	0.33 **	0.17 *	0.24 **	0.11	0.12	0.15	0.15 *	−0.01	---					
12. Attention C/M	−0.04	0.42 **	0.20 **	0.16 *	0.24 **	0.23 **	0.18 *	0.17 *	0.11	0.08	0.09	---				
13. Clarity C/F	0.23 **	0.16 *	0.38 **	0.20 **	0.05	−0.04	0.08	0.20 **	0.16 *	0.13	0.28 **	0.15 *	---			
14. Clarity C/M	−0.03	0.33 **	0.46 **	0.09	0.21 **	0.21 **	0.13	0.27 **	0.17 *	0.15	0.29 **	0.27 **	0.51 **	---		
15. Repair C/F	0.34 **	0.14	0.17 *	0.27 **	−0.10	−0.09	0.07	0.11	0.20 **	0.18 *	0.19 *	0.17 *	0.60 **	0.23 **	---	
16. Repair C/M	0.10	0.18 *	0.14	0.35 **	−0.08	0.05	−0.09	0.03	−0.18 *	0.18 *	0.17 *	0.07	0.17 *	0.36 **	0.25 **	---
Mean	4.22	3.43	3.28	3.27	3.44	3.40	3.37	3.36	3.43	3.28	2.87	3.45	3.28	3.56	3.12	3.20
S.D.	0.90	0.80	0.74	0.77	0.81	0.83	0.82	0.78	0.81	0.73	0.79	0.79	0.79	0.72	0.92	0.84

* *p* < 0.05, ** *p* < 0.01; C = Children; F/C = Perceived EI of fathers about children: M/C = Perceived EI of mothers about children; C/F = Perceived EI of children about fathers; C/M = Perceived EI of children about mothers.

**Table 2:** Regression coefficients, standard errors, and model summary information for the serial multiple mediator model with EI perceived by parents about children as explanatory variables (model related to H1).

Consequent												
	M1 (Attention Child)			M2 (Clarity Child)			M3 (Repair Child)			Y (MH5)		
Antecedent	Coeff.	*SE*	*p*	Coeff.	*SE*	*p*	Coeff.	*SE*	*p*	Coeff.	*SE*	*p*
Constant	1.9170	0.3801	0.0000	2.0795	0.3923	0.0000	2.4394	0.4411	0.0000	3.2815	0.5270	0.0000
Attention F/C	0.2936	0.0852	0.0007	0.1448	0.0847	0.0894	−0.0655	0.0887	0.4545	0.0271	0.0973	0.7808
Attention M/C	0.1619	0.0842	0.0564	−0.0556	0.0818	0.4978	−0.1341	0.0850	0.1166	−0.1386	0.0938	0.1416
Clarity F/C	−0.1391	0.0991	0.1624	0.0054	0.0957	0.5704	−0.0361	0.0994	0.7170	−0.1447	0.1089	0.1860
Clarity M/C	0.0780	0.0995	0.4343	0.1972	0.0957	0.0409	0.0767	0.1006	0.4473	0.0206	0.1104	0.8525
Repair F/C	0.1048	0.0917	0.2548	−0.0862	0.0883	0.3304	−0.0832	0.0920	0.3669	0.2976	0.1010	0.0037
Repair M/C	−0.0603	0.0941	0.5228	−0.0121	0.0905	0.8942	0.1840	0.0939	0.0519	−0.0017	0.1041	0.9870
M1	---	---	---	0.1108	0.0752	0.1424	0.2530	0.0920	0.1166	−0.2851	0.0862	0.0012
M2	---	---	---	---	---	---	0.2530	0.0816	0.0023	0.1529	0.0920	0.0982
M3	---	---	---	---	---	---	---	---	---	0.3675	0.0863	0.0000
	*R*^2^ = 0.1610 *F*(6,163) = 5.2130; *p* = 0.0001			*R*^2^ = 0.1026 *F*(7,162) = 2.6455; *p* = 0.0129			*R*^2^ = 0.1158 *F*(8,161) = 2.6359; *p* = 0.0097			*R*^2^ = 0.2299 *F*(9,162) = 5.3067; *p* = 0.0000		

(F/C = Perceived EI of fathers about children; M/C = Perceived EI of mothers about children).

**Table 3:** Indirect and direct effects for the mediation model using EI perceived by parents about children as explanatory variables.

Y = MH5 Child	Specific Indirect Effects							Total Indirect Effect	Direct Effect
X	Mediator 1 (Attention Child)	Mediator 2 (Clarity Child)	Mediator 3 (Repair Child)	Mediator 4 (Att -> Cl)	Mediator 5 (Att -> Rep)	Mediator 6 (Cl -> Rep)	Mediator 7 (Att -> Cl -> Rep)		
Attention F/C	−0.0837 (−0.1726; −0.0199)	0.0221 (−0.0153; 0.0739)	−0.0244 (−0.1149; 0.0348)	0.0050 (−0.0030; 0.0243)	0.0071 (−0.0089; 0.0323)	0.0135 (−0.0035; 0.0448)	0.0030 (−0.0010; 0.0120)	−0.0574 (−0.1726; 0.0483)	0.0271 (−0.1651; 0.2194) (*p* = 0.7808)
Attention M/C	−0.0461 (−0.1099; 0.0003)	−0.0085 (−0.0528; 0.0208)	−0.0493 (−0.1390; 0.0150)	0.0027 (−0.0018; 0.0136)	0.0039 (−0.0052; 0.0199)	−0.0052 (−0.0303; 0.0105)	0.0017 (−0.0006; 0.0075)	−0.1008 (−0.2164; −0.0091)	−0.1386 (−0.3238; 0.0467) (*p* = 0.1416)
Clarity F/C	0.0396 (−0.0147; 0.0984)	0.0083 (−0.0329; 0.0676)	−0.0133 (−0.0923; 0.0686)	−0.0024 (−0.0118; 0.0020)	−0.0034 (−0.0187; 0.0048)	0.0051 (−0.0237; 0.0287)	−0.0014 (−0.0067; 0.0009)	0.0326 (−0.0710; 0.1334)	−0.1447 (−0.3598; 0.0704) (*p* = 0.1860)
Clarity M/C	−0.0222 (−0.0864; 0.0330)	0.0302 (−0.0158; 0.0971)	0.0282 (−0.0505; 0.1105)	0.0013 (−0.0029; 0.0090)	0.0019 (−0.0051; 0.0145)	0.0183 (−0.0017; 0.0570)	0.0008 (−0.0015; 0.0050)	0.0584 (−0.0454; 0.1674)	0.0206 (−0.1975; 0.2386) (*p* = 0.8525)
Repair F/C	−0.0299 (−0.0927; 0.0271)	−0.0132 (−0.0665; 0.0194)	−0.0306 (−0.1013; 0.0389)	0.0018 (−0.0027; 0.0098)	0.0025 (−0.0065; 0.0143)	−0.0080 (−0.0298; 0.0123)	0.0011 (−0.0013; 0.0054)	−0.0763 (−0.1793; 0.0299)	0.2976 (0.0982; 0.4970) (*p* = 0.0037)
Repair M/C	0.0172 (−0.0378; 0.0808)	−0.0018 (−0.0442; 0.0383)	0.0676 (0.0007; 0.1508)	−0.0010 (−0.0082; 0.0034)	−0.0015 (−0.0123; 0.0064)	−0.0011 (−0.0234; 0.0214)	−0.0006 (−0.0049; 0.0017)	0.0787 (−0.0224; 0.1923)	−0.0017 (−0.2073; 0.2039) (*p* = 0.9870)

F/C = Perceived EI of fathers about children; M/C = Perceived EI of mothers about children; Att = Attention; Cl = Clarity; Rep = Repair.

**Table 4:** Regression coefficients, standard errors, and model summary information for the serial multiple mediator model with EI perceived by children about parents as explanatory variables (model related to H2).

Consequent												
	M1 (Attention Child)			M2 (Clarity Child)			M3 (Repair Child)			Y (MH5)		
Antecedent	Coeff.	*SE*	*p*	Coeff.	*SE*	*p*	Coeff.	*SE*	*p*	Coeff.	*SE*	*p*
Constant	0.7703	0.3590	0.0334	1.0373	0.3427	0.0029	1.3775	0.3800	0.0004	3.2603	0.4563	0.0000
Attention C/F	0.2795	0.0699	0.0001	0.1998	0.0690	0.0043	0.0811	0.0763	0.2891	0.0776	0.0884	0.3817
Attention C/M	0.3567	0.0687	0.0000	0.0942	0.0698	0.1793	0.1505	0.0758	0.0486	−0.0149	0.0885	0.8669
Clarity C/F	−0.0950	0.0954	0.3208	0.1763	0.0901	0.0520	0.0698	0.0983	0.4789	0.0789	0.1137	0.4887
Clarity C/M	0.2072	0.0945	0.0297	0.3283	0.0902	0.0004	−0.2533	0.1012	0.0133	−0.1580	0.1192	0.1867
Repair C/F	0.0233	0.0737	0.7521	−0.0517	0.0694	0.4574	0.1117	0.0750	0.1384	0.2634	0.0872	0.0029
Repair C/M	0.0407	0.0686	0.5542	−0.0249	0.0647	0.7009	0.3249	0.0698	0.0000	0.0104	0.0859	0.9033
M1	---	---	---	−0.0472	0.0737	0.5229	−0.0860	0.0796	0.2820	−0.3047	0.0923	0.0012
M2	---	---	---	---	---	---	0.2182	0.0848	0.0109	0.1248	0.0999	0.2133
M3	---	---	---	---	---	---	---	---	---	0.2678	0.0910	0.0037
	*R*^2^ = 0.3056 *F*(6,163) = 11.9543; *p* = 0.0000			*R*^2^ = 0.2855 *F*(7,162)= 9.2488; *p* = 0.0000			*R*^2^ = 0.2397 *F*(8,161) = 6.3453; *p* = 0.0000			*R*^2^ = 0.2638 *F*(9,160) = 6.3706; *p* = 0.0000		

(C/F = Perceived EI of children about fathers; C/M = Perceived EI of children about mothers).

**Table 5:** Indirect and direct effects for the mediation model using EI perceived by children about parents as explanatory variables.

Y = MH5 Child	Specific Indirect Effects							Total Indirect Effect	Direct Effect
X	Mediator 1 (Attention Child)	Mediator 2 (Clarity Child)	Mediator 3 (Reparation Child)	Mediator 4 (Att -> Cl)	Mediator 5 (Att -> Rep)	Mediator 6 (Cl -> Rep)	Mediator 7 (Att -> Cl -> Rep)		
Attention C/F	−0.0851 (−0.1608; −0.0242)	0.0249 (−0.0169; 0.0892)	0.0217 (−0.0188; 0.0826)	−0.0016 (−0.0133; 0.0063)	−0.0064 (−0.0234; 0.0054)	0.0117 (0.0000; 0.0385)	−0.0008 (−0.0047; 0.0029)	−0.0357 (−0.1299; 0.0806)	0.0776 (−0.0970; 0.22522) (*p* = 0.3817)
Attention C/M	−0.1087 (−0.2228; −0.0394)	0.0118 (−0.0135; 0.0578)	0.0403 (−0.0083; 0.0960)	−0.0021 (−0.0143; 0.0099)	−0.0082 (−0.0052; 0.0199)	−0.0052 (−0.0291; 0.0091)	0.0055 (−0.0060; 0.0215)	−0.0010 (−0.0053; 0.0050)	−0.0149 (−0.1897; 0.1600) (*p* = 0.8669)
Clarity C/F	0.0289 (−0.0374; 0.0927)	0.0220 (−0.0208; 0.0788)	0.0187 (−0.0408; 0.0857)	0.0006 (−0.0028; 0.0063)	0.0022 (−0.0032; 0.0133)	0.0103 (−0.0021; 0.0362)	0.0003 (−0.0013; 0.0023)	0.0829 (−0.0374; 0.0927)	0.0789 (−0.1456; 0.3035) (*p* = 0.4887)
Clarity C/M	−0.0631 (−0.1377; 0.0068)	0.0410 (−0.0304; 0.1368)	−0.0678 (−0.1642; −0.0047)	−0.0012 (−0.0117; 0.0041)	−0.0048 (−0.0204; 0.0039)	0.0192 (0.0000; 0.0585)	−0.0006 (−0.0042; 0.0018)	−0.0774 (−0.2077; 0.0724)	−0.1580 (−0.3933; 0.0773) (*p* = 0.1867)
Repair C/F	−0.0071 (−0.0587; 0.0498)	−0.0065 (−0.0416; 0.0208)	0.0299 (−0.0120; 0.0958)	−0.0001 (−0.0044; 0.0018)	−0.0005 (−0.0070; 0.0044)	−0.0030 (−0.0171; 0.0084)	−0.0001 (−0.0017; 0.0007)	0.0126 (−0.0715; 0.1194)	0.2634 (0.0911; 0.4357) (*p* = 0.0029)
Repair C/M	−0.0124 (−0.0661; 0.0353)	−0.0031 (−0.0422; 0.0221)	0.0870 (0.0176; 0.1738)	−0.0002 (−0.0031; 0.0029)	−0.0009 (−0.0078; 0.0039)	−0.0015 (−0.0175; 0.0088)	−0.0001 (−0.0011; 0.0014)	0.0687 (−0.0342; 0.1707)	0.0104 (−0.1591; 0.1800) (*p* = 0.9033)

C/F = Perceived EI of children about fathers; C/M = Perceived EI of children about mothers; Att = Attention; Cl = Clarity; Rep = Repair.
